# Psora—Its Nature

**Published:** 1895-03

**Authors:** Frank W. Patch

**Affiliations:** South Framingham, Mass.


### PSORA—ITS NATURE.

FRANK W. PATCH, M. D., SOUTH FRAMINGHAM, MASS.

MR. CHAIRMAN AND MEMBERS OF THE BŒNNINGHAUSEN CLUB:—It is with considerable hesitation that this modest paper is presented to the members of the club to-night, not only on account of a vivid memory of the valuable contribution read at the last meeting, but also with a certain awe of the magnitude of this subject which the revered Hahnemann spent so many years in elucidating, and which he finally left imprinted at every line with the image of his vast intuitive and empirical knowledge.

At best I can only endeavor to lay before you a condensed picture of the position which this miasm occupies, theoretically and practically, in the mind of the homœopathic physician of to-day as gleaned chiefly from the writings of Hahnemann and his co-workers.

Since his time so little has been added to the literature of the subject, and so many of his self-styled followers have drifted from even a knowledge of the first principles of the doctrine as taught by the master, that we find it hardly worth the time needed to con the general homœopathic writings of our present period in the expectation of securing anything of value.

At the outset let us inquire into the nature of our subject. What is its genesis? in fact, what are we to understand by the term psora?

The ancient Greek writers define it as "any cutaneous disease attended with abundant exudation, pustulation, and crusting." Hahnemann classes it as a "chronic miasm," yet Foster defines "miasm" as "a morbific emanation which affects individuals directly—*i. e.*, not through the medium of another individual." This, however, we do not understand to be always a condition of psora. Hahnemann evidently held a different view of the meaning of the term in question. He distinctly states that he designates the name psora, "a general term" (*Chronic Diseases*, p. 21, Vol. I), intended to apply to the "internal enemy" or "disease;" "a sort of internal itch, which may exist either with or without an eruption upon the skin."

After long observation and study of the nature of disease, Hahnemann concluded that all chronic disease originated in the three miasms, syphilis, sycosis, and psora, the last being the fountain-head of all chronic, non-venereal ailments except those caused by bad medication or the fumes of certain minerals.

It seems evident that Hahnemann found that far back in the early time, long before the history of mankind began to be recorded, there had sprung up on earth what might be termed the spirit of virulent, self-developing miasmatic disease, known only by its successive groups of symptoms, and changing with each cycle of its appearance.

This disease essence or ego had, throughout all time, perpetuated itself in varying forms, yet always with the same death-like grasp had held its numberless victims.

At one time the dynamic disturbance had manifested itself as a malignant itch, at another as leprosy; again, "the plague,"

small-pox, and so on. In Hahnemann's own time the itch was its most prominent form, and who shall say that the recent epidemic influenza is not the latest evidence of the hold which this hydra-headed monster still has upon the inhabitants of earth.

To this disease ego Hahnemann has applied the name of *psora*.

This seems to us the broadest interpretation which it is possible to apply to the term and the one most strongly borne out in the writings of our esteemed master.

The fact of the non-acceptance of Hahnemann's theories of chronic disease by so great a part of the homœopathic world to-day may be, in part, due to the somewhat general feeling that his teachings refer solely to the itch, which a superficial reading of his writings might imply, as the source of the multitude of non-venereal chronic symptoms met with in practice.

That Hahnemann did not so intend to teach we feel most confident, and that the time of his birth may have had something to do with the prominent position which the itch occupied in his theories of disease is more than probable. During, and for many years prior to his time, scabies was prevalent to a great extent among all classes of people. It was considered by the majority of physicians then, as to-day, a purely local skin disease (*Chronic Diseases*, p. 130, Vol. I) now thought, additionally, to have its source in the acarus and to end with the extinction of this offender.

The presence of the parasite has been so easy of demonstration, so very evident to all observers, that the anti-Hahnemannian here found one of his most bland arguments against the dynamic origin of *all* diseases as elucidated by our first teacher. To those who elect to follow his lead, however, this seeming mountain proves no stumbling-block, for would we not as soon seek the true cause of gonorrhœa in the gonococcus as that of scabies in the acarus? This point of view illustrates the value of a working basis of natural law, in that each of these affections falls into our well-known channels of knowledge without confusion or debate.

We all understand, it is true, that the animal parasite, the acarus, is a nearly constant accompaniment of the disease in question, but as a result rather than cause, and it is equally true that the death of the parasite by germicidal methods is *not* the end of the disease, monstrous authority to the contrary notwithstanding.

Hahnemann announced his discovery of *Similia* in 1796; published his *Organon* in 1810, but it was not until 1827, in his seventy-third year, that he announced to his followers what he considered his most important discovery—the "Theory of the nature of chronic disease." Indissolubly incorporated with this discovery was the doctrine of *psora*. Instead of, as is often told us, this discovery being an hallucination of his second childhood, it would seem to the impartial student of his life that it was the product of his ripest years of thought and experience, being a direct succession to the production of the *Organon*. The causes leading to this discovery may be gleaned from his writings, as when we read that "Ever since the years 1816 and 1817, I had been employed day and night to discover the reason why the homœopathic remedies which were then known did not effect a true cure of the above-named chronic diseases (non-venereal). I tried to obtain a more correct, and, if possible, a completely correct idea of the true nature of those thousands of chronic ailments which remained uncured, in spite of the incontrovertible truth of the homœopathic doctrine" (*Chronic Diseases*, p. 18, Vol. I).

Hahnemann observed "that a previously existing itch was the cause why many diseases that appeared to be separate and coherent maladies should not be cured by homœopathic treatment." "All the subsequent sufferings were dated from the time when the psoric eruption had manifested itself."

"These circumstances, coupled with the fact that psoric eruptions which had been removed by evil practices or by some other cause were evidently followed in otherwise healthy persons by chronic ailments having the like or similar symptoms, left me no doubt about the internal enemy which I had to combat in my medical treatment" (*Chronic Diseases*, p. 20, Vol. I).

Hahnemann arrived at this conclusion after years of study and application, and the repeated observation of the failure of the ordinary homœopathic remedies to effect satisfactory cures in the host of chronic non-venereal diseases.

He says: "I observed that the non-venereal chronic diseases, even after having been repeatedly and successfully removed by the then known homœopathic remedies, continually reappeared in a more or less modified form, and with a yearly increase of disagreeable symptoms. This proved to me the fact that the phenomena which appeared to constitute the ostensible disease ought not to be regarded as the whole boundaries of the disease—otherwise the disease would have been completely and permanently cured by homœopathic drugs, which was not the case; but that this ostensible disease was a mere fragment of a much more deep-seated, primitive evil, the great extent of which might be inferred from the new symptoms which continued to appear from time to time. This showed me that the homœopathic practitioner ought not to treat diseases of this kind as separate and completely developed maladies; nor that he ought to expect such a permanent cure of these diseases as would prevent them from appearing again in the system, either in their original or in a modified and often more disagreeable form. I became convinced that the first condition of finding out one or more homœopathic medicines which should cover all the symptoms characterizing the whole disturbance was *to discover all the ailments and symptoms inherent in the unknown primitive malady.* The medicine being found out, the physician would then be able to conquer and completely to extinguish the whole disease, together with its successively appearing groups of symptoms. This primitive disease evidently owed its existence *to some chronic miasm*" (*Chronic Diseases*, p. 19, Vol. I).

"Psora," he says, "is the oldest, most universal and most pernicious chronic miasmatic disease; the oldest history of the oldest nations does not reach its origin." "Unless it is thoroughly cured, it lasts until the last breath of the longest life. Its secondary symptoms have become innumerable. All chronic ailments now existing, which have not been produced by bad medical treatment, or by the fumes of quicksilver, lead, arsenic, etc., originated in psora as their fountain head." Hahnemann finds records of psora among the annals of the oldest peoples. The "Israelites," the "Greek barbarians," and the "Arabs" were infected in one or another manner. The uncivilized peoples of the Middle Ages were not less diseased, and the frightful plagues that visited Europe at different periods and from varying causes, malignant erysipelas, leprosy, etc., etc.—he attributes to the rage of the same disease force.

Now, in the light of our present knowledge, just what conclusions are we to reach concerning the application of this term "psora"? a name that meant so much to the first Hahnemannians, and that plays so important a part in our nomenclature of to-day. Frankly, we deem that the word conveyed a far broader meaning to the mind of Hahnemann than a simple symbol for the acarus poisoning, as some of our friends would fain have us believe, or even the result of the repellance of the itch. For instance, he says at one time that "the psoric eruption which appeared after infection had taken place, and which, in civilized countries, had been reduced to a simple manifestation of the common itch, was easily driven from the skin by all sorts of contrivances." Again: "During the centuries when the psoric eruption was first known in the form of leprosy, patients, though they suffered much in consequence of lancinating pains in the tumors and scabs and the vehement itching all around, enjoyed, nevertheless, a fair share of general health" (*Chronic Diseases*, p. 26, Vol I). And further: "The milder forms of psora which appeared again during the fourteenth and fifteenth centuries in the shape of the itch infected a far greater number than the leprous patients were able to do, whose frightful appearance caused them to be avoided by everybody" (*Chronic Diseases*, p. 27, Vol. I). In speaking of the comparative results of leprosy and the itch Hahnemann says: "There is another disadvantage, which is this, that the essence of this reduced psora (the itch) is unchanged; that it is equally formidable as before, and that, being more easily repelled from the skin, it appears so much more imperceptibly upon the inner surfaces. The chief symptom, which is the external eruption, having been suppressed, it produces an innumerable quantity of secondary chronic ailments" (*Chronic Diseases*, p. 27, Vol. I).

In his long observation of chronic diseases Hahnemann learned that some undiscovered principles underlay the whole subject; that a natural law, more far-reaching than any yet taught, governed the appearance and disappearance of chronic complaints. He saw in the successive inroads of plague after plague during the previous centuries a connecting influence of causation which rendered it impossible for him to accept each as a passing circumstance.

In Hahnemann's time and for the previous half-century or more, scabies was a very prevalent disease in Europe. All physicians found ample opportunity for observation of its characteristics. Hahnemann, in common with others, noted the evil effects of suppression of the eruption, and details many cases in proof.

But he alone connects the itch disease with other manifestations of the so-called psora which had existed from time immemorial in varying forms.

Judging from several passages in the *Chronic Diseases*, we feel that Hahnemann recognized a difference between the prevalent "common itch" of his own day and that which he mentions once or twice as a "malignant itch" occurring in ancient times, though this difference was but in degree, as he elsewhere states that "names are of no consequence here, since the essence of this miasmatic itch is everywhere the same" (*Chronic Diseases*, p. 25, Vol. I).

We cannot understand that Hahnemann intended to teach that *all* secondary or latest psoric symptoms *were* the direct result of the suppression of the itch disease, as witness what Grauvogl says on this subject:

"Whoever made clinical studies at a time when the itch was to be found spread over all parts of the cutaneous surface, except the face and genitalia, and had only been suppressed by external means, has observed very frequently, even during this external treatment, the sudden occurrence of fatal inflammation of the brain or lungs, diffuse gout, dropsy, etc. At the same time, no other cause could be found for this than the change of all the functions of the cutaneous surface by the itch and its injurious treatment.

"Thus, he had even the sensible perception thereof, yet thought as little of pronouncing the itch mite the *only* cause and condition of these diseases as he would declare the operation for rectal fistula as the only cause and condition of the tuberculosis following it" (*Text-Book of Homœopathy*, p. 272).

Physicians of this vicinity see, at the present time, comparatively little of the itch, nor have they for many years; yet there is no apparent subsidence of psora. It seems that at certain periods of earth's history psora has presented what might be termed *grand* manifestations. These upheavals vary according to the conditions of the life and environment of the time, both as regards character and severity. As examples of this theory, we would mention leprosy, which Hahnemann classes as a psoric disease, and which he says was so prevalent in the year 1226 that there were in France about two thousand houses for the reception of patients. Again, we have small-pox, at one time the most prevalent disease. At the period in which Hahnemann lived the itch was the centre of attention, and it is probable that we have recently passed through still another minor *grand* period of psoric eruption, as before stated, in the character of the epidemic influenza of the past five years.

These prominent periods of disease would seem to correspond in nature somewhat to other phases of evolution, a cogent example of which may be seen even in the animal kingdom, where we find a continual change going on as time proceeds.

There was a period, we are told, when egg-layers were the predominant animal race; then came the marsupials, overrunning the earth in great abundance, and, still later, the saurians.

Now, at the present day egg-laying animals are reduced to a few families, but, at the same time, have evolved into another branch—the bird family. Of the intermediate group, the marsupials, we have left only the kangaroo, while the saurians also are nearly extinct, and so on; yet there are *still to be found*, even at this late day, examples of each of these ancient periods of prominence, just as we find remnants of past races in the Zuñi and like peoples left behind in the whirl of evolution. The point we desire to illustrate is this: that after any great periodic upheaval of psoric disease we are not to expect comparative disappearance of the particular disease in question under a long period of time, nor complete immunity under a great many years, if at all.

That certain diseases are now prevalent is not an argument against their psoric character or their periodic position in the cycle of disease.

Even now we have on earth examples of animal families practically extinct long before the beginning of the history of man, just as we still meet examples of disease which were at their zenith of psoric disturbance before the history of medicine was thought of. These are not seen at present, however, in great or epidemic prevalence, but as sporadic cases, limited epidemics or endemic to certain localities. Just what proportion of influence in the disappearance of the *grand* manifestations of psoric disease is due to police regulations and boasted discovery of peculiar methods of causing artificial immunity, and what to the fact of the subsidence of the telluric or other dynamic disturbance that created them, would be most interesting to know.

Our great mistake in the study of disease as a whole is, as Hahnemann often pointed out, the separating of its every manifestation into disconnected segments, and forgetting its dynamic origin as well as its greater consecutive nature and successive development. We attempt to cure the particular condition in hand, having little regard for the spiritual ego, without which the disease would be *nil*.

What a subtle connection is there binding together the seeming vagaries of the life force! We know that all disease originates in the vital centres, not only because Hahnemann said it, but because, when we stop to think, we know that everything on earth is dynamic in origin, and that dynamic laws control every vibration of life from the atom upward. What we need is to discover the action of these laws if we would reach the seat of disease, instead of wasting time on less important questions.

Psora, the "mother" of all chronic non-venereal diseases, as stated by Hahnemann, is always, in its original attack, accompanied by an eruption or other manifestation on the surface of the body which appears within a few days after infection; if it take the form of the itch disease, it may, we are told, be speedily cured with one or two doses of homœopathically prepared Sulphur. But if the internal disease is neglected, and attention directed simply to suppression of the eruption by harsh, external measures alone, which in this case may easily be done, we have brought about a condition of "latent psora" which stands ready at all times to break forth into a long line of secondary symptoms or diseases, which often keep up a sort of hide-and-seek with health as long as life lasts. The exact character of these symptoms is governed chiefly by the constitution and environment of the patient, and it is from among their number that most of the chronic diseases which are met in daily practice are drawn.

This matter of the evil results of suppression seems to be an axiom of disease life. Its gravitation toward the surface of the body can be disturbed no more than that of steam in a boiler, as is evident not only in the *grand* periodic upheavals of psoric disease, but also in those miasmatic troubles classed as secondary psoric conditions by Hahnemann. This is the case with intermittent fever, which, he says, will never attack a person not under psoric influence. We know, moreover, that the same law holds good in the acute diseases characterized by eruptive processes, and also that the evil results following such suppression are often prolonged for many years, rivaling, in severity and depth of disturbance of the vital forces, psora itself. And yet, strange as it may seem, we are still taught that in *chronic* diseases suppression is the chief end to be sought, and this state of affairs will probably hold sway until it is possible to institute investigations and compile statistics covering long enough periods of time to prove these facts to the mathematical majority of the profession.

The patience of a Hahnemann is as essential to-day as ever if we expect to see our tenets become the recognized law of all. In searching for outside reference to Hahnemann's theory of chronic diseases, some interesting remarks by Grauvogl were brought to light, in which he complains of the "schemata" of Hahnemann, in that "these form too much of a chaos, they lack that precision of form which would enable one to infer the law underlying them. But," he goes on to say, "that here these phenomena *are* controlled by a natural course of events from given elements, admits of no doubt." The same versatile author says that Dr. Reuter, of Nuremberg, "declares he has observed in chronic diseases, during his practice of many years, stages, like those which mark acute diseases, in the various forms of the reciprocal action of the acarus poison within the organism. He gives the following characteristics as regards the succession of stages in diseases arising therefrom, provided that up to the last stage no medical aid had been sought: 1. Gastroses; 2. Catarrhs; 3. Hemorrhoids; 4. Sweat of the feet; 5. Hoarseness; 6. Headache and toothache; 7. Diseases of the eyes; 8. Diseases of the ears; 9. Prurigo of the trunk, Furunculosis; 10. Swelling of the cervical glands; 11. Rheumatisms; 12. Swelling of the axillary glands.

"His experience indicates to him an aggravation of the general constitutional status, if, after a disease from among those named under these numbers had passed by, another of a higher number makes its appearance.

"The whole series, for the most part, refers to adult age; and if, for example, one suffered from chronic inflammation of the eyes, it was highly probable that the chronic ailments from 1 to 7 had been present in previous years.

"In like manner he takes it to be an extension of the acarus disease; if, after that inflammation of the eyes, even though it had been cured, there should subsequently appear diseases of the ears, prurigo, rheumatism, or swelling of the cervical or axillary glands" [*Text-book of Homœopathy*, part II, p. 228].

The same author further says:

"Since I am, moreover, obliged for the easier comprehension of chronic diseases to mention those thereof which, according to Hahnemann, contain the productive stage of his psora, and because Hahnemann simply enumerated them without any grounds of classification, I am induced, by Reuter's observation, to introduce them according to the above-named succession, for it contains, at any rate, a leading principle for further investigation."

That there is some basis of fact in these observations of Reuter and Grauvogl is more than possible, and they may sometime serve as a beginning of further investigations along the same line.

It would seem as though the case records of those who examine patients according to the rules laid down by Hahnemann should throw much light upon the subject.

The glory of Homœopathy in general, and the fame of any Hahnemannian in particular, must ever rest in great part on superior results in the treatment of chronic diseases, and we should push ahead in this field with all vigor.

One of the chief arguments of those who oppose the doctrine of psora is that "Mr. Blank never had the itch; we *know it*." Granted! Yet we contend that he may be a victim of psoric poison, for the secondary symptoms of this dread condition seem to be directly transmissible through we know not how many generations.

The point is not only whether a given patient has ever been treated by external, violent measures for scabies, or had a leprosy sore suppressed, but is he afflicted with psoric disease, as evidenced by any chronic, intractable ailment or group of secondary psoric symptoms laid down by Hahnemann in his masterly work on *Chronic Diseases*. If so we may feel very confident that our patient either in his own person or that of some progenitor has been a victim of one of the periodic grand manifestations of ancient psora, the most persistent miasm of which we have record. Knowing this, if we value our patient's future condition of health, and our own professional reputation, let us endeavor to give him the benefit of the best antipsoric treatment of which we can attain knowledge.

